# Draft Genome Sequences of Two Marine Phototrophic Bacteria, *Erythrobacter longus* Strain DSM 6997 and *Erythrobacter litoralis* Strain DSM 8509

**DOI:** 10.1128/genomeA.00677-14

**Published:** 2014-07-24

**Authors:** Yu Wang, Rui Zhang, Qiang Zheng, Nianzhi Jiao

**Affiliations:** State Key Laboratory of Marine Environmental Science, Institute of Marine Microbes and Ecospheres, Xiamen University, Xiamen, People’s Republic of China

## Abstract

Aerobic anoxygenic phototrophic bacteria (AAPB) are important functional groups and are widely distributed in the global upper ocean. Here we report the draft genomic sequences of two marine AAPB isolates belonging to the genus *Erythrobacter*, *Erythrobacter longus* strain DSM 6997 and *Erythrobacter litoralis* strain DSM 8509.

## GENOME ANNOUNCEMENT

Aerobic anoxygenic phototrophic bacteria (AAPB) play significant roles in the carbon cycle in the oceans (1). They are obligate aerobes and perform photoheterotrophic metabolism (2). AAPB contain bacteriochlorophyll *a* (BChl *a*), are widely distributed in the class *Proteobacteria*, and are closely related to purple photosynthetic bacteria (3–5). The first isolate of marine AAPB was *Erythrobacter longus* DSM 6997 (also named OCh 101) (6). Later, more species of AAPB were isolated from diverse marine environments (3, 7). Recently, the genome of another type strain, *Erythrobacter* sp. strain NAP1, has been sequenced, which does not contain genes involved in CO_2_ or N_2_ fixation that confirmed the photoheterotrophy of the AAPB (8).

*Erythrobacter longus* strain DSM 6997, isolated from the surface of green seaweed *Enteroporpha linza*, is a type strain of the *Erythrobacter* genus (3). The strain DSM 6997 is characterized by Gram-negative and orange-pigmented rods (6) and has the ability to store polyhydroxyalkanoate (PHA) as a carbon source in the cell (9). Moreover, another species, *Erythrobacter litoralis* strain DSM 8509, was isolated from a cyanobacterial mat sample (10). The strains DSM 6997 and DSM 8509 are both slightly halophilic and multiply by binary division (10). Here we report the draft genomes of these two type strains of the *Erythrobacter* genus.

The draft genomes of these two strains were obtained by Illumina mate-paired sequencing technology. Mate-paired reads of average 100-bp length were assembled using Velvet software (v1.2.03) (11). Total contig sizes of ~3.55 Mbp with an average of 680× coverage of strain DSM 6997 and ~3.17 Mbp with an average of 780× coverage of strain DSM 8509 were obtained. The overall G+C contents of strains DSM 6997 and DSM 8509 are 57.37% and 65.15%, respectively. The open reading frames (ORFs) were identified using GLIMMER (12). Gene prediction was performed using BLASTn against the non-redundancy (nr) nucleotide database to identify the orthologous sequences. A total of 3,078 protein-coding genes were obtained in the genome of strain DSM 6997 and 2,793 genes were obtained in strain DSM 8509. tRNAscan-SE was employed to identify the tRNA sequences (13), followed by rRNA identification performed using RNAmmer software (14). There are 3 rRNAs and 42 tRNAs and 4 rRNAs and 44 tRNAs in the genomes of strains DSM 6997 and DSM 8509, respectively.

Gene annotation was performed using BLASTn against the nr database and KEGG protein database (15). There are 2,023 proteins and 2,118 proteins that annotated to clear functions in the genomes of strains DSM 6997 and DSM 8509, respectively. The functions of the genes are defined by association with clusters of orthologous groups (COG) classification against the conserved domains database (CDD) (16) and the KEGG pathway collection (17). There are 2,281 proteins that are classified to COG categories in the genome of strain DSM 8509 and 2,329 proteins are assigned to different COG categories in the DSM 6997 genome. Relatively high similarities of DSM 6997 and DSM 8509 to *Erythrobacter* sp. NAP1 are observed.

### Nucleotide sequence accession numbers.

These whole-genome shotgun projects have been deposited at DDBJ/EMBL/GenBank under the accession no. JMIW00000000 and JMIX00000000 for strain DSM 6997 and strain DSM 8509, respectively. The versions described in this paper are versions JMIW01000000 and JMIX01000000.
